# Eosinophilic gastroenteritis; a report of two cases with different presentations 

**Published:** 2017

**Authors:** Amir Sadeghi, Elham Abdi, Negin Jamshidfar, Farnoush Barzegar, Farhad Lahmi

**Affiliations:** 1 *Gastroenterology and Liver Diseases Research Center, Research Institute for Gastroenterology and Liver Diseases, Shahid Beheshti University of Medical Sciences, Tehran, Iran*; 2 *Foodborne and Waterborne Diseases Research Center, Research Institute for Gastroenterology and Liver Diseases, Shahid Beheshti University of Medical Sciences, Tehran, Iran*; 3 *Basic and Molecular Epidemiology of Gastrointestinal Disorders Research Center, Research institute for Gastroenterology and Liver Diseases, Shahid Beheshti University of Medical Sciences, Tehran, Iran*; 4 *Behbood Gastroenterology and Liver Diseases Research Center, Shahid Beheshti University of Medical Sciences, Tehran, Iran*

**Keywords:** Eosinophilic gastroenteritis, Presentation, Gastrointestinal disorders

## Abstract

Eosinophilic gastroenteritis is a rare inflammatory disease, defined by infiltration of eosinophils in gastrointestinal (GI) tract, but the etiology of this disorder is unknown. Depends on the involvement region of Eosinophilic gastroenteritis, GI symptoms are variable including abdominal pain, malabsorption, gastric and duodenal ulcer. Due to its non-specific symptoms, the diagnosis is based on upper GI endoscopy followed by histopathological examination of the biopsies, which shows eosinophilic infiltration in different layers of GI tract. In this article we report two cases with gastrointestinal disorders. The first case was a 52-year-old man with a history of peripheral edema over the past 3 months and low level of serum albumin. All the necessary evaluations were done and increase number of eosinophils were found in duodenal biopsies. The second case was a 42-year-old man presented with a history of chronic diarrhea over the past two years. Main causes of diarrhea were ruled out and small intestine biopsies confirmed submucosal eosinophilic infiltration. Therefore, corticosteroid therapy was administered for both patients then they were followed for a year. During this time all of the symptoms were disappeared and they did not recur in the first year of follow up.

## Introduction

 Eosinophilic gastrointestinal disorder (EGIDs) is an inflammatory disease which is defined as a primary increasing of eosinophils in gastrointestinal tract without a known cause (1). This disease affects gastrointestinal tract from esophagus to rectum, but the most involved sites are stomach and small intestine (2). Depending on the location and the involved layer, the clinical presentations of this disease are variable. According to the Kleins proposal, (3) the disease is classified into three types including mucosal involvement, muscular involvement, and serosal involvement. Mucosal involvement is the most predominant type, presents with malabsorption, bleeding, and protein losing enteropathy. Gastric and duodenal ulcers are the other manifestations of mucosal involvement (4-7). Moreover, mucosal involvement is the most associated type to food intolerance or allergy in more than 50% of patients (8). 

EGIDs occur at both sexes, but male is slightly predominant. This disorder affects any age with the peak in 3^th^ or 4^th^ decades of the life (18). Patients, especially children, with atopic disorders, food intolerance or allergies are more susceptible for EGIDs (9).

As Peripheral eosinophilia is found in most of the patients, it is not a necessary criterion for the diagnosis of EGIDs (10,11). Besides, high level of Immunoglobulin E (IgE) is found in the serum of some patients. Primary eosinophilic cationic protein (ECP) and substances such as major basic protein I and II are some biomarkers that exist in the granules of the eosinophils and determine the activity of the disease. (12). Therefore, the most accepted ways for the diagnosis of EGIDs is based on the manifestation of gastrointestinal symptoms, the result of histopathological examination (more than 20 power-high field), and exclusion of other causes of eosinophilia (2). Food allergy- a part of the atopic syndrome- represents an adverse immune response toward certain food proteins which causes the infiltration of eosinophils in gastrointestinal tract (13).

There is no food allergy test for determining the role of foods in clinical improvement of EG symptoms or tissue eosinophilia. However, food allergy is the significant reason of disease in some cases, so the first step of treatment is a diet without pathogenic food (14). However, The study showed that the certain treatment of disease is 2-week corticosteroid therapy (prednisolone 20-40 mg/d)(15,16).

## Case presentation

**Case1**

A 52 years old man referred to the gastroenterology clinic with a history of peripheral edema in lower limbs over the past 3 months. Any correlative complaint like abdominal pain, nausea, vomiting, fever or weight loss are not presented. His past medical history was unremarkable for any diseases or injuries and no symptoms of food or drug allergy or atopy were reported.

He was eupneic and afebrile and upon physical examination, his vital signs were normal. The result of cardiac examination was normal and no evidence of abnormal bowel sounds or palpable mass or visceromegaly were detected on abdominal examination. A Laboratory investigations showed in the table 1.

No Ova/parasites were noted on repeated stool examinations. Also no remarkable findings were detected in ultrasonography. Additionally, computed tomography of abdomen and pelvis was performed and revealed thickening of jejunal and ileal walls and increased density of mesenteric fat. At the next step, he underwent upper endoscopy and colonoscopy. The results showed no abnormality, however the duodenal biopsy showed increased number of eosinophil (30/ per high power field). Therefore, the diagnosis of EGID was made and prednisolone therapy was administered at a dose of 30 mg daily. Moreover, diet restriction was started. After 2 weeks he was clinically improved and discharged. After 4 weeks follow up, the lower limbs edema disappeared, and blood protein, albumin and eosinophil count became normal. He was followed for one year and the disorder did not recur.

**Table 1:** Laboratory investigations

Hemoglobin	12.1gr/dl
Leukocyte	5800/mm3
Eosinophils	430/mm3
Platelet	430000/mm3
ESR	28mm/hr
CRP	5.2mg/L
ALT	116U/L
AST	102U/L
ALP	630U/L
Serum albumin	2.5gr/dl
Serum protein	4.9U/L
Ferritin	15
Anti TTG IgA	Normal
Total IgA	Normal

**Case 2**

A 42-year-old man with a history of chronic diarrhea over 2 years was admitted to our clinic. He did not complain of any GI symptoms such as abdominal pain, nausea, weight loss or nocturnal diarrhea and also there was not a history of food allergy or atopy.

Upon physical examinations, nothing was significant. Abdominal examination was normal. The results of laboratory tests are presented in table 2.

Liver function tests were normal and repeated stool exam for ova/parasite and serological test for toxocara and strongyloides were negative.

For the evaluation of malignancy, the patient underwent the bone marrow aspiration and biopsy which results were negative. Workup for chromosomal testing including evaluation of BCR-ABL mutation and *Jack2* mutation was subsequently negative. The results of abdominal CT and small bowel transmit were normal. As a result of hematological workup and absence of organ damage, the diagnosis of hypereosinophilic syndrome was excluded. After that, upper endoscopy was performed which showed severe erythematous and giant duodenal folds. Histological evaluation of duodenal and antral biopsies demonstrated dense submucosal eosinophilic infiltration. Prednisolone therapy was started on the dose of 20mg daily. A few days after the initiation of therapy, the symptoms of the patient were improved and steroid therapy was maintained for three weeks. After the cessation of the therapy, eosinophil count dropped and his symptoms were disappeared completely. He was followed for 1 year and remained asymptomatic. 

**Table 2:** Laboratory results

Hemoglobin	14.5gr/dl
Leukocyte	5480/mm3
Eosinophils	1790/mm3
Serum albumin	3.7gr/dl
Total protein	6.3gr/dl
Anti TTG IgA	1.3

## Discussion

Eosinophilic gastroenteritis is a rare inflammatory disease, characterize by eosinophil infiltration in GI tract in the absence of underlying disease such as tissue-invasive helminth, atopy, hematological malignancies, solid tumors, vasculitis, infectious disease, connective tissue disorders, and drug reactions such as tacrolimus, and enalapril. (17-19). We present two cases with different complaints from peripheral edema in lower limbs and chronic diarrhea respectively. Peripheral edema is a nonspecific finding with variable etiologies such as Heart failure, nephrotic syndrome, cirrhosis, hypoproteinemia and drug reactions. (20) In case 1, nothing was remarkable in medical history and physical examinations but the laboratory findings indicated blood eosinophilia with normal leukocytes count and high numbers of inflammatory markers and hypoalbuminemia. Peripheral eosinophilia can be found in most of the patients with EG, its presence is not necessary for diagnosis (10,11). Therefore, the patient underwent ultrasonography and computed topography for the diagnosis of hypoalbuminemia and inflammations but the only significant findings were thickening of jejunal and ileal walls and increased density of mesenteric fat. However, the diagnosis is strongly based on GI endoscopy and colonoscopy and histopathological examinations which demonstrate excessive eosinophils infiltration in one or more segments of GI tract (2). 

Case 2 describes chronic diarrhea during 2 years and blood eosinophilia without any relative sign or symptom. The etiologies of chronic diarrhea include inflammatory bowel disease (IBD), infectious diseases, ischemic colitis, radiation colitis and neoplasia (21). The most important differential diagnosis is neoplasia which should be evaluated by bone marrow aspiration and biopsy, and chromosomal testing. In the absence of any disease and either, existence of eosinophils infiltration in the submucosal layer of GI tract the EG is diagnosed. Corticosteroid therapy is efficient for the treatment of all types of EG, so both patients were administered on corticosteroid therapy and followed for 1 year. During that time the evidence of recurrence did not appear. However, it should be noticed that recurrence of the disease is common after the therapy. Hence, low-dose maintenance prednisolone therapy is effective for those with a more relapsing disorder (15,16). Moreover, it is reported that in patients with EG who are dependent on corticosteroid therapy montelukast, a selective and competitive leukotriene receptor antagonist, is an efficient drug of treatment (22). Tan et al. 2001 (23) reported a patient with EG who was treated with non-enteric coated budesonide because of less side effects in comparison with corticosteroid therapy.

Eventually, for the treatment of GI obstruction which is limited to one segment of GI tract, bowel surgical resection is indicated. But we should know that the recurrence of the disease in the other segment is common, so medical treatment is indicated after surgery (24-26).

## Conflict of interests

The authors declare that they have no conflict of interest.
